# Exosomal LINC01213 Plays a Role in the Transition of Androgen-Dependent Prostate Cancer Cells into Androgen-Independent Manners

**DOI:** 10.1155/2022/8058770

**Published:** 2022-03-10

**Authors:** Zhuifeng Guo, Xuwei Lu, Fan Yang, Chang He, Liang Qin, Ning Yang, Conghui Han, Jiawen Wu

**Affiliations:** ^1^Department of Urology, Minhang Hospital, Fudan University/Minhang Branch, Zhongshan Hospital, Fudan University, Shanghai, China; ^2^Medical College of Soochow University, Suzhou, Jiangsu Province, China; ^3^Department of Urology, Xuzhou Central Hospital, Xuzhou, Jiangsu Province, China

## Abstract

**Background:**

Castration-resistant prostate cancer (CRPC), one of the prostate cancers, is a medical conundrum around the world. Some studies have demonstrated that many long noncoding RNAs in exosomes are very important in many types of cancer, including prostate cancer. However, until now, the function of exosomes in the occurrence and development of CRPC has not been reported.

**Methods:**

In vitro, cell coculture was used in LNCap cells and PC-3 cells, while the isolation and purification of exosomes and the subsequent treatment assays were used in functional studies. In vitro assays were performed to detect the transformation of ADPC cells (androgen-dependent prostate cancer) into AIPC cells (androgen-independent prostate cancer). Subsequently, a lncRNA-sequencing assay was performed to detect different lncRNA expression profiles in ADPC cells cocultured with or without AIPC exosomes. The role of LINC01213 was analysed by a TCGA database after silencing the expression of LINC01213. CCK-8, qRT-PCR, and Western blotting studies were performed to analyse the possible mechanism by which exosomes participate in prostate cancer progression.

**Results:**

In the coculture system, ADPC cells acquired androgen deprivation tolerance through exosome-mediated intercellular communication. Exosomes secreted by AIPC cells can promote the transformation of ADPC cells into androgen-independent cells in vitro and in vivo. lncRNA sequencing showed that LINC01213 was upregulated in exosomes derived from AIPC cell lines. The rescue experiments were preformed, and the results revealed that most of the functions of LINC01213 were performed by Wnt/*β*-catenin.

**Conclusions:**

All the findings showed that exosomes play a key role in CRPC progression by upregulating LINC01213 and activating Wnt/*β*-catenin signalling.

## 1. Introduction

Prostate cancer (PCa) is one of the most common malignancies among men in the world [1]. In China, both incidence and death rates from prostate cancer have also increased significantly over the past decade [2]. Although many treatments have been used to treat prostate cancer, such as radical resection and radiotherapy [3], the therapeutic effect is not very satisfactory; most patients eventually revert to castration-resistant prostate cancer (CRPC) [3]. Hence, finding out the pathogenesis of CRPC and further studying its mechanism is the key step to solve this problem, which also is the goal of the research.

Exosomes are extracellular vesicles (EVs) that have been used as a new therapy tool, including drug delivery and antitumor therapy, for a variety of diseases [4]. Almost all cells release exosomes [5], including normal and tumor cells [6, 7]. Exosomes are even found in blood [8], urine [9], and saliva [10]. Exosomes contain not only proteins but also multiple different types of RNA molecules, lncRNAs are included [11], and exosomes also act as messengers between cells that carry messages to each other [5, 12]. The study of exosomes in prostate cancer is increasing day by day, and the function of exosomes in prostate cancer has been reported by many researchers. It has been shown that lncRNA-p21 is present in exosomes of PCA patients, and this level of lncRNA-p21 may be helpful for improving the diagnosis and prediction of the malignant state in PCA patients [13]. Zhang et al. reported that in prostate cancer, exosomes can activate heme oxygenase-I to affect the progression of prostate cancer [14].

Noncoding long RNA is a new kind of noncoding RNA, which is more than 200 nucleotides (nT) in size and does not encode any protein, and the main reason is that they lack an open reading framework [15–17]. Current studies suggest that lncRNAs can be implicated in various human cancers, and there have been reports of breast cancer [18], lung cancer [19], and gastric cancer [20]. With respect to prostate cancer, there are also related reports, for example, PCA3 and SChLAP1 were reported to be biomarkers for prostate cancer [21–23]. PCGEM1 and PRNCR1 play a key role in androgen-dependent transcription by promoting chromatin cyclization between the AR-binding enhancer and promoter of target gene [24]. In addition, a previous study confirmed that LINC01213 plays an important role in cancers [25, 26]; Until now, the function of LINC01213 is in its infancy of prostate research.

The concept of the tumor microenvironment has been proposed in recent years, and many researchers have focused on the role of tumor microenvironment in the development and progression of tumors, including prostate cancer [27]. Therefore, tumor microenvironment may be involved in the androgen resistance process. We are interested in exosomes that play a key role in cell-cell interactions. The purpose of this study was to investigate the exosomal role of LINC01213 in the transformation of androgen-dependent prostate cancer cells into androgen-independent prostate cancer cells.

## 2. Materials and Methods

### 2.1. Cell Culture

The prostate cancer cell lines LNCaP and PC-3 were purchased from the American Type Culture Collection (ATCC; Rockville, MD, USA). RPMI1640 medium was supplemented with 10% fetal bovine serum (FBS, Gibco, Grand Island, NY, USA) and 1% penicillin streptomycin (Gibco). All cells were cultured in 5% CO_2_ incubator humidified at 37°C.

### 2.2. Coculture of LNCAP Cells with PC-3 Cells

LNCaP cells and PC-3 cells were cocultured in the transwell coculture system (0.4 *μ*m transwell insert). LNCaP cells seeded in superior cavity and PC-3 cells were cocultured in inferior cavity for 10 days. When PC-3 cells reached 90% confluence, LNCAP cells were transferred to a new plate.

### 2.3. Exosomes Isolated by Ultracentrifugation

In order to remove the dead cells, we centrifuged the harvested cultures (300 ml) in 500 g for 30 min and then continued centrifuging in 2000 g for 30 min. After that, the supernatant was collected and centrifuged at 10000 g for 30 min, collected the supernatant again, and centrifuged at 100000 g for 120 min for obtaining exocrine granules. At this step, the pellet was collected and resuspended in PBS.

### 2.4. lncRNAs Sequencing

We used the miRNeasy kit (Qiagen) to extract total RNA from DHT-treated LNCaP cells. RNA mass was measured by the Agilent Bioanalyzer. Then, according to the published scheme [27], all samples were sequenced with Illumina HiSeq 2000 (read length 100 nT) according to the previous reports [28].

### 2.5. CCK-8 Determination

Cell proliferation was detected with the CCK-8 kit (Kumamoto Dojindo, Japan). Seed the cells in the 96-well plate at 1000/well density. After 0, 1, 2, 3, and 4 days, 10 L CCK-8 (5 mg/ml) was added to each well and incubated at 37°C for 2 hours. Finally, the cells were measured at 450 nm with an enzyme analyzer (ThermoFisher Scientific, Waltham, MA, USA).

### 2.6. Cell Cycle Assay

The transfected cells were dissolved overnight in precooled ethanol (75%) at 4°C, washed with cold PBS once, and stained with BD, Pharmingen^TM^, PI/RNase at room temperature for 30 min. Finally, cell cycles (GO/G1, S, and G2) were analysed by flow cytometry (BD Biosciences).

### 2.7. Tumorigenicity Assay

Male NOD/SCID mice (Shanghai Bikai) aged 4–6 weeks were used to determine their tumorigenicity. The experimental design was approved by the Local Ethics Committee of Minhang Hospital of Fudan University (certificate no.: 2020-032-01K). Before the androgen ablation experiment in vivo, we castrated the mice carefully. After that, two groups of cells were injected into mice; one group cell was treated with PBS, while another one was treated with exosomes. After 40 days, the mice were killed, and the tumors were removed.

### 2.8. Real-Time Quantitative PCR (qRT-PCR)

Total RNA was extracted from cells using TRIzol reagent (15596026, Invitrogen, Carlsbad, CA, USA). For qRT-PCR, the HiScript II first strand cDNA synthesis kit (Vazyme Biotech Co.) was used generate the first station cDNA. Gene expression was detected with SYBR ex Taq premix (RR420A, Takara, Takara, Dalian, China). All PCRs were detected by at least three copies, and the results were standardized by GAPDH, which were calculated using the 2^−ΔΔCT^ method. The gene specific primers were designed and synthesized by SprinGen Biotech, and their sequences are given in Supplementary Table S1.

### 2.9. Small Interfering RNA (siRNA) Transfection

The effective siRNA targeting human LINC01213 was purchased from RiboBio (Guangzhou, China). Lipofectamine 2000 (11668019, Invitrogen, Carlsbad, CA, USA) was used to transfect siRNA, and the final transfection concentration was 10 nM. Finally, qRT-PCR was used to detect the expression changes.

### 2.10. Western Blotting

The cells were scraped off with a spatula and then lysed with RIPA buffer which was added with protein inhibitors. The BCA protein assay kit (Beyotime, China) was used to determine the protein concentration. The total protein (30 ug) or exosomes were then isolated by SDS-PAGE gel and transferred to PVDF membrane (1620177, PVDF, BioRad, Hercules, USA). The membranes were sealed in 5% skim milk for 60 min and then treated at 4°C with primary antibodies (PSA, AR, TSG101, HSP70, and Alix) overnight; all primary antibodies dilution ratios are 1–1000. On the second day, the membranes were clear with PBS for 3 times, and the second antibody was then incubated at room temperature for 1 hour. Finally, ELC luminescence was used for measurement the protein bands.

### 2.11. Statistical Analysis

All experiments were made in triplicate, and the data were analysed using GraphPad Prism 8 (CA, USA) software. All data were expressed as mean ± standard deviation (SD). Student' *t*-test was used to estimate the significance of the difference between the two groups, and one-way analysis of variance (ANOVA) was used to compare the two groups. The statistical significance was *P* < 0.05(^*∗*^) and *P* < 0.01(^*∗∗*^), respectively.

## 3. Result

### 3.1. Androgen Deprivation Resistance Induced by Coculture of LNCaP Cells and PC-3 Cells

**Figure 1:**
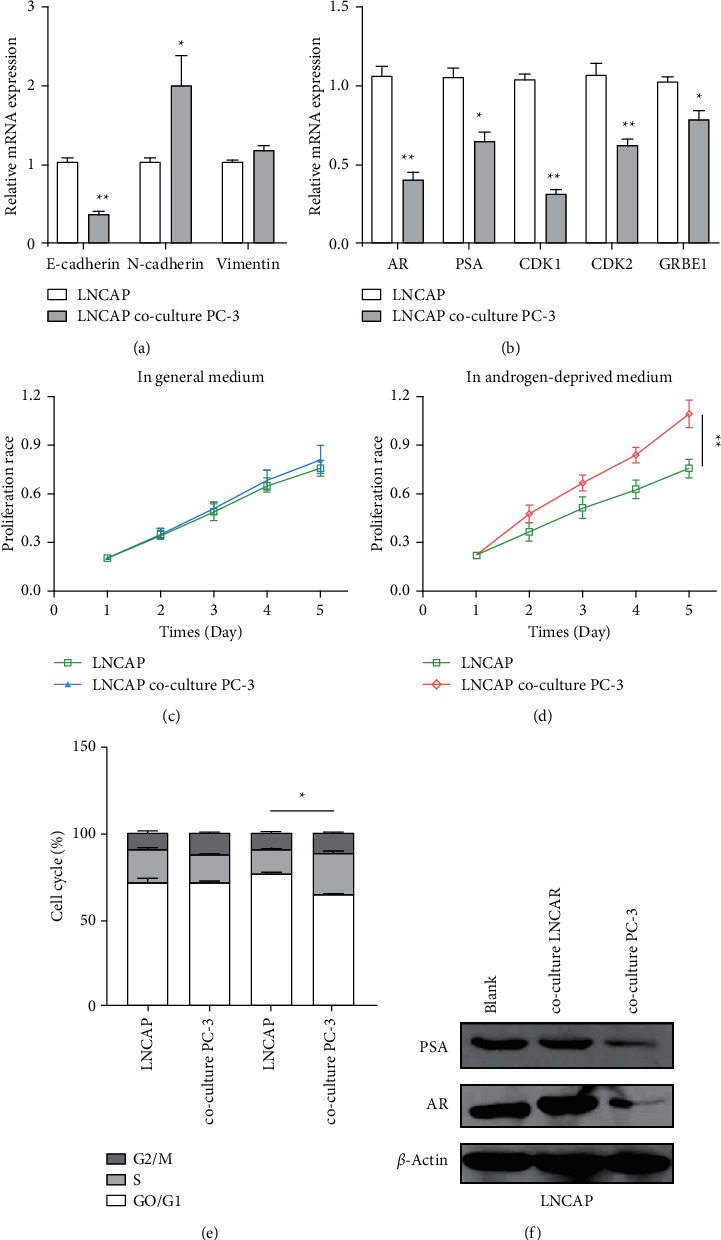
The change of resistance of LNCaP cells in coculture with PC-3 cells. (a)-(b) qRT-PCR used to detect the gene level of E-cadherin, N-cadherin, vimentin, AR, PSA, CDK1, CDK2, and GRBE1 after treatment. (c)-(d) CCK-8 used to detect the proliferation of LNCaP cells and PC-3 cells in normal and androgen media. (e) The cell cycle of LNCaP cells and PC-3 cells cocultured on conventional or androgen medium was detected. (f) The protein level of PSA and AR in cocultured LNCAP/PC-3 cells analysed by Western blot. ^*∗*^*P* < 0.05, ^*∗∗*^*P* < 0.01.

To investigate trend resistance, we choose two cells for this study: one is LNCaP cell and another is PC-3. First, LNCAP cells and PC-3 cells were cocultured in a transwell system, LNCaP cells were cultured in lower cavity, and PC-3 cells were cultured in upper cavity. After 10 days, we found that AR-responsive genes and EMT marker genes were significantly changed after coculturing; CDK1, CDK2, and GRBE1 were downregulated, and N-cadherin was upregulated, while E-cadherin was downregulated in LNCAP cells (Figures 1(a) and 1(b)). Compared with the normal medium control group, the proliferation of LNCaP cells in the androgen-deficient medium group increased significantly (Figures 1(c) and 1(d)). The results of flow cytometry showed that the S phase was significantly increased in the cocultured group under castration conditions, but there was no significant difference under normal conditions. Western blotting assay was performed to detect the protein level of AR and PSA, and the result showed that both AR and PSA were significantly decreased in the coculture condition (Figures 1(e) and 1(f)).

### 3.2. Isolation and Identification of Exosomes

**Figure 2:**
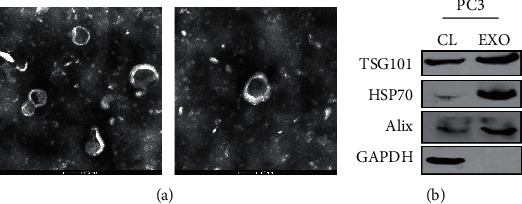
The characterization of isolated exosomes. (a) Exosomes isolated from PC-3 cells identified by electron microscopy. (b) Western blot analysis of exosome markers. CL, cell lysate; EXO, exosome.

Why do LNCaP cells have such a great influence on coculture? We suspected that the exosomes of PC-3 cells played a key role, so the exosomes were isolated from the culture supernatant of PC-3 cells. Transmission electron microscopy showed that the appearance of membrane-limited particles was uniform and ranged from approximately 100 nm. Western blotting of exosomal markers such as Alix, TSG101, and HSP70 showed that the granules had exosomal characteristics and could be separated uniformly (Figures 2(a) and 2(b)).

### 3.3. Exosomes Derived from AIPC Cells Promote Emasculation Resistance in ADPC Cells

**Figure 3:**
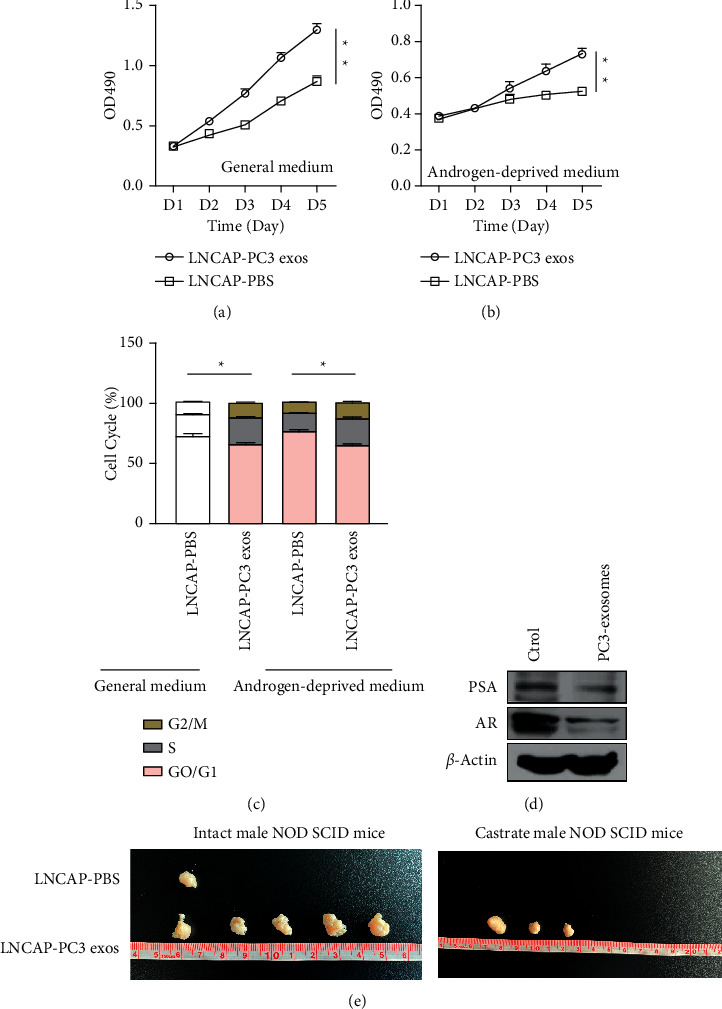
The role of exosomes derived from PC-3 in castration resistance of LNCaP cells. (a)-(b) Proliferation of exosomes-treated LNCaP cells and PC-3 cells in normal and androgen media evaluated with CCK-8. (c) Flow cytometry used to detect the cell cycle of LNCaP cells and PC-3 cells treated with exosomes in the conventional medium and androgen medium. (d) The expression of PSA and AR in PC-3 exocrine analysed by Western blot. (e) Tumorigenesis experiment showing that LNCaP cells cocultured with PC-3 exosomes had stronger tumorigenesis ability, especially under castration condition. ^*∗*^*P* < 0.05, ^*∗∗*^*P* < 0.01.

In order to further investigate the role of PC-3 exosomes in the castration resistance of LNCaP cells, we first incubated PC-3 exosomes (50 g/ml) with LNCaP cells, and then, CCK-8 assay and flow cytometry assay were used to detect the proliferation and cell cycles. After incubating PC-3 exosomes, the proliferation of LNCaP cells increased significantly not only in androgen-deprived medium but also in general medium (Figures 3(a) and 3(b)). Similar results occurred in the cell cycles assay; after incubating PC-3 exosomes, the S phase of LNCaP cells increased in both different condition (Figure 3(c)), and the protein levels of PAR and PAR decreased significantly after treated with PC-3 exosomes (Figure 3(d)).

In subsequent studies, animal experiments were performed with intact and castrated NOD/SCID mice. The tumorigenesis data showed that, after treated with PC-3 exosomes, the tumorigenicity of LNCaP cells was significantly higher than that of normal LNCaP cells. It should be noted that normal LNCaP cells cannot form tumors under castration conditions, but some mice injected with the treated LNCaP cells have the ability to form tumors (Figure 3(e)), suggesting that AIPC cells may be induced by androgen secretion.

### 3.4. LINC01213 May Play a Key Role in AIPC-Derived Exosomes in ADPC Cells

**Figure 4:**
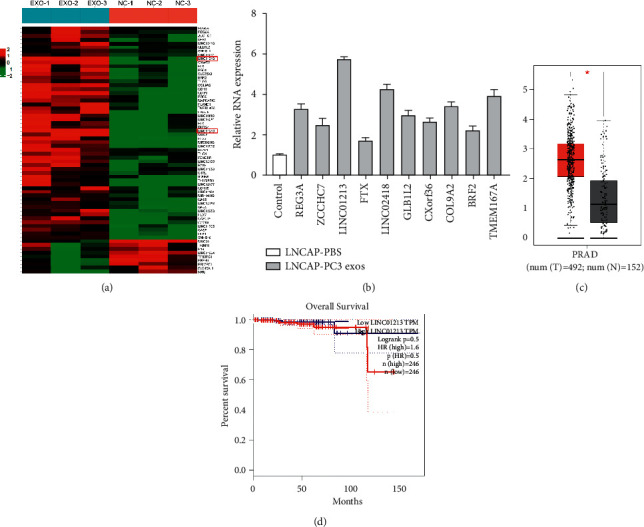
Exosome PC-3 upregulates LINC01213, which may play a key role in prostate cancer. (a) Heatmap showing all differentially expressed ncRNA between LNCaP cells cocultured with PC-3 exosomes and control cells. (b) qRT-PCR analysis confirmed the expression of first 10 lncRNA. (c) LINC01213 levels in 492 PRAD and 152 normal tissues on the GEPIA website. (d) Kaplan–Meier survival analysis (quartile of the two sets of criteria) performed on the prognosis of 246 patients with PAC, which is available on the GEPIA website (the criteria of the two groups were quartile).

There are many substances in exosomes, such as microRNA, lncRNA, or circRNA. To study the underlying mechanism of exosome-induced castration resistance, we focused on lncRNA. We performed the lncRNA-sequencing assay; a heatmap of the lncRNA expression profiles showed 61 lncRNAs were found to be significantly affected (1.5-fold change), with 52 lncRNAs increased and 9 decreased (Figure 4(a)). KEGG pathway analysis showed that the most affected ncRNAs were those involved in tumor-related pathways, such as the Wnt/-catenin pathway (Supplementary Figure 1).

The first 10 lncRNAs were verified by qRT-PCR. In order to evaluate the role of LINC01213 in prostate cancer, we first analysed the expression of LINC01213 in the TCGA database and found that it was upregulated in prostate cancer. We also found that the expression of LINC01213 correlated with prognosis of prostate cancer, and the higher the expression, the worse the prognosis (Figures 4(c) and 4(d)).

### 3.5. LINC01213 Inhibits the Growth of ADPC Cells through Wnt/-Catenin Signal Transduction and Partially Reduces Androgen Dependence

**Figure 5:**
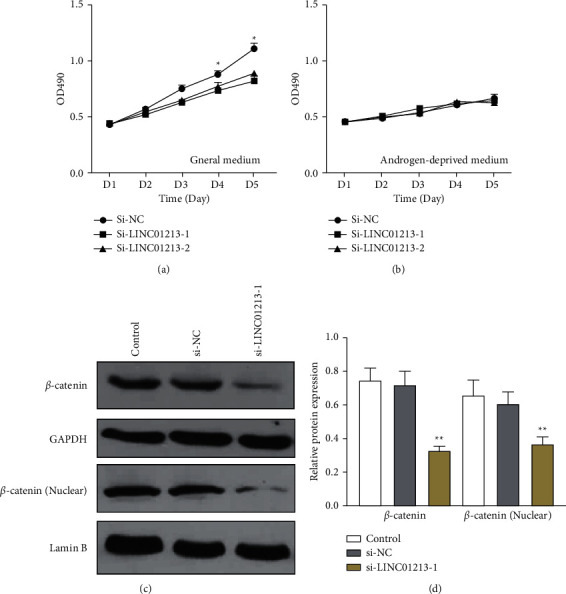
LINC01213 enhances androgen-independent behaviour via Wnt/*β*-catenin signalling. (a)-(b) Cell growth of si-NC/si-LINC01213 LNCAP cells in conventional and androgen media. (c)-(d) Protein levels of total and nuclear *β*-catenin in wild-type (control) LNCAP cells and cells transfected with si-NC or si-LINC01213. Data are shown as the mean ± standard deviation (SD) from three independent experiments. ^*∗*^*P* < 0.05, ^*∗∗*^*P* < 0.01.

In order to study the androgen-dependent effect of LINC01213 on ADPC cells, LINC01213 was downregulated by siRNA, and CCK-8 assay was used to detect the prefoliation again, and the result showed that the prefoliation of LNCaP cells was inhibited under normal conditions (Figure 5(a)), and they cannot proliferate in the absence of androgen (Figure 5(b)). Our results further showed that downregulating LINC01213 in LNCAP cells significantly decreased *β*-catenin protein levels (Figures 5(c) and 5(d)).

## 4. Discussion

The development of prostate cancer is a complex process, and the transition from androgen-dependent to androgen-independent is an even more complex process; sometimes, ADPC cells and AIPC cells can coexist in a certain period of time. Therefore, exosomes may play a key role for this transformation. Our results suggest that coculture with PC-3 cells in androgen medium can promote the proliferation of LNCaP cells, but how PC-3 cells endow LNCaP cells with castration resistance is still unclear.

Exosomes are 40–100 nanometre-sized vesicles that are released into the extracellular space from many cell types. In the human body, various body fluids contain these vesicles [5, 29, 30]. Exosomes affect the phenotype of recipient cells through intercellular communication. Until now, there are few studies on exosomes. In recent years, researchers have found that exosomes play multiple roles in many diseases, such as inflammation [31], and there have been many studies on exosomes in cancer, for example, the potential use of exosomes as biomarkers for the diagnosis and treatment of breast cancer [12]. Exosome-mediated miR-200b promotes the proliferation of colorectal cancer [32]. In prostate cancer, Husseini-Behesti et al. reported that prostate cancer-derived excretion promotes proliferation and migration of LNCaP cells [33]. Zhang et al. reported that in prostate cancer, exosomes can activate heme-oxygenase-I to affect the progression of prostate cancer [14]. Nevertheless, there are less reports that focus on function of exosomes in prostate cancer, especially in the transformation. In the current research, our results showed the role of exosomes not only in vitro but also in vivo. The results showed that after treated with PC-3 exosomes, the tumorigenicity of LNCAP cells was markedly enhanced. Normal LNCAP cells could not grow under castrated conditions, but with the treatment of PC-3 exosomes, cells had tumor-forming ability. These results show that exosomes play a key role in the process of the transformation.

In order to further elucidate the mechanism of castration resistance induced by AIPC exosomes, we used RNA-seq to detect the differential expression of lncRNA in LNCaP cells before and after PC-3 exosomes treatment. The results of KEGG indicated that the differentially expressed lncRNAs are associated with the regulation and cancer-related pathways such as the Wnt/*β*-catenin pathway.

There are two kinds of ncRNA: short ncRNA and long noncoding RNA (lncRNAs) [34]. lncRNAs have been confirmed to participate in the regulation of tumorigenesis and its progression into tumor suppressor genes or oncogenes [35, 36], including prostate cancer [37]. LINC01213 is a long noncoding intergenic RNA, which has been reported in breast cancer [26]. We found that the expression of LINC01213 was overexpression in prostate cancer. At the same time, upregulation of LINC01213 activated the Wnt/*β*-catenin pathway. Taken together, our findings suggest that LINC01213 may be a novel therapeutic target for CRPC patients.

To sum up, our study reveals the existence of LINC01213 molecule in exosomes of prostate cancer cells for the first time. Our findings may help to clarify the role of tumor microenvironment in the development of CRPC and provide a new direction for the treatment of CRPC.

## Data Availability

The datasets used and/or analysed during the current study are available from the corresponding author upon request.

## Supplementary Materials

Supplementary Materials Supplementary Figure 1. KEGG analysis of the differentially expressed lncRNAs. Supplementary Table 1. List of the primer sequences for PCR.
