# Supplementary material for: Exosomal LINC01213 Plays a Role in the Transition of Androgen-Dependent Prostate Cancer Cells into Androgen-Independent Manners
Source: J Oncol. 2022 Mar 10;2022:8058770. doi: 10.1155/2022/8058770 (PMC8930242; doi:10.1155/2022/8058770)
Supplement: Supplementary Materials — Supplementary Figure 1. KEGG analysis of the differentially expressed lncRNAs. Supplementary Table 1. List of the primer sequences for PCR. [file 8058770.f1.zip › 8058770.f1/Supplementary Table S1.pdf]

Table 1 List of the Primer Sequences for PCR

Target	Sequence (5`-3`)
AR	Forward: CCAGGGACCATGTTTGCC<br>Reverse: CGAAGACGACAAGATGGACAA
PSA	Forward: TGTCCGTGACGTGACGTGCATTGG<br>Reverse: GCCAGGGTTGGGAATGCTT
CK1	Forward: GGATGTGCTTATGCAGGATTCC<br>Reverse: CATGTACTGACCAGGAGGGATAG
CK2	Forward: CCAGGAGTTACTTCTATGCCTGA<br>Reverse: TTCATCCAGGGGAGGTACAAC
GRBE1	Forward: ATGGGGAAATTCTTACGCTGGAC<br>Reverse: CACTCGGCTACCACCTTCT
E-cadherin	Forward: ATTTTTCCCTCGACACCCGAT<br>Reverse: TCCCAGGCGTAGACCAAGA
N-cadherin	Forward: AGCCAACCTTAACTGAGGAGT<br>Reverse: GGCAAGTTGATTGGAGGGATG
Vimentin	Forward: AGTCCACTGAGTACCGGAGAC<br>Reverse: CATTTCACGCATCTGGCGTTC
GAPDH	Forward: ACAGCCTCAAGATCATCAGCAAT<br>Reverse: ATGGACTGTGGTCATGAGTCCTT
REG3A	Forward: GCAATGGACTCAATCAACAA<br>Reverse:TTACGAATGGCCAGTGCGTA
ZCCHC7	Forward: CTTATTAGGGACCCATCCGT<br>Reverse: AATCCATCGGCATCGACATG
LINC01213	Forward: ACACGTCTGGGAGAAAGCAA<br>Reverse: TGGTTTCTCCCAATTTAGG
FTX	Forward: GGCCAATGCATTTGAATA<br>Reverse: GGTACAATTCGGAGTTCCAGT
LINC02418	Forward: CAGAAGTGTGAGCCCACTGT<br>Reverse: TGGCATTTTCAGTCTTCTTC
GLB1L2	Forward: GTAAGTCCCTAGTCACCCA<br>Reverse: CCATTCAAGTCCAGTCAACC
CXorf36	Forward: AATTGGGGCACAAATGGCT<br>Reverse: ATTCAAGGGACTTAGTAACG
COL9A2	Forward: GGATTACCACGTTGCCGTAA<br>Reverse: AAATCCATTGGCACCGTGGC
BRF2	Forward: CCTTGCAGTTAAGTCCCGAT<br>Reverse: GCCGTTAAACGTCAAATTAGA
TMEM167A	Forward: TTCCAAGGCTTCGTAAGCCT<br>Reverse: GGACTTGCCCAGTTAAGTCT
