# Supplementary figures and images for: Exosomal LINC01213 Plays a Role in the Transition of Androgen-Dependent Prostate Cancer Cells into Androgen-Independent Manners
Source: J Oncol. 2022 Mar 10;2022:8058770. doi: 10.1155/2022/8058770 (PMC8930242; doi:10.1155/2022/8058770)

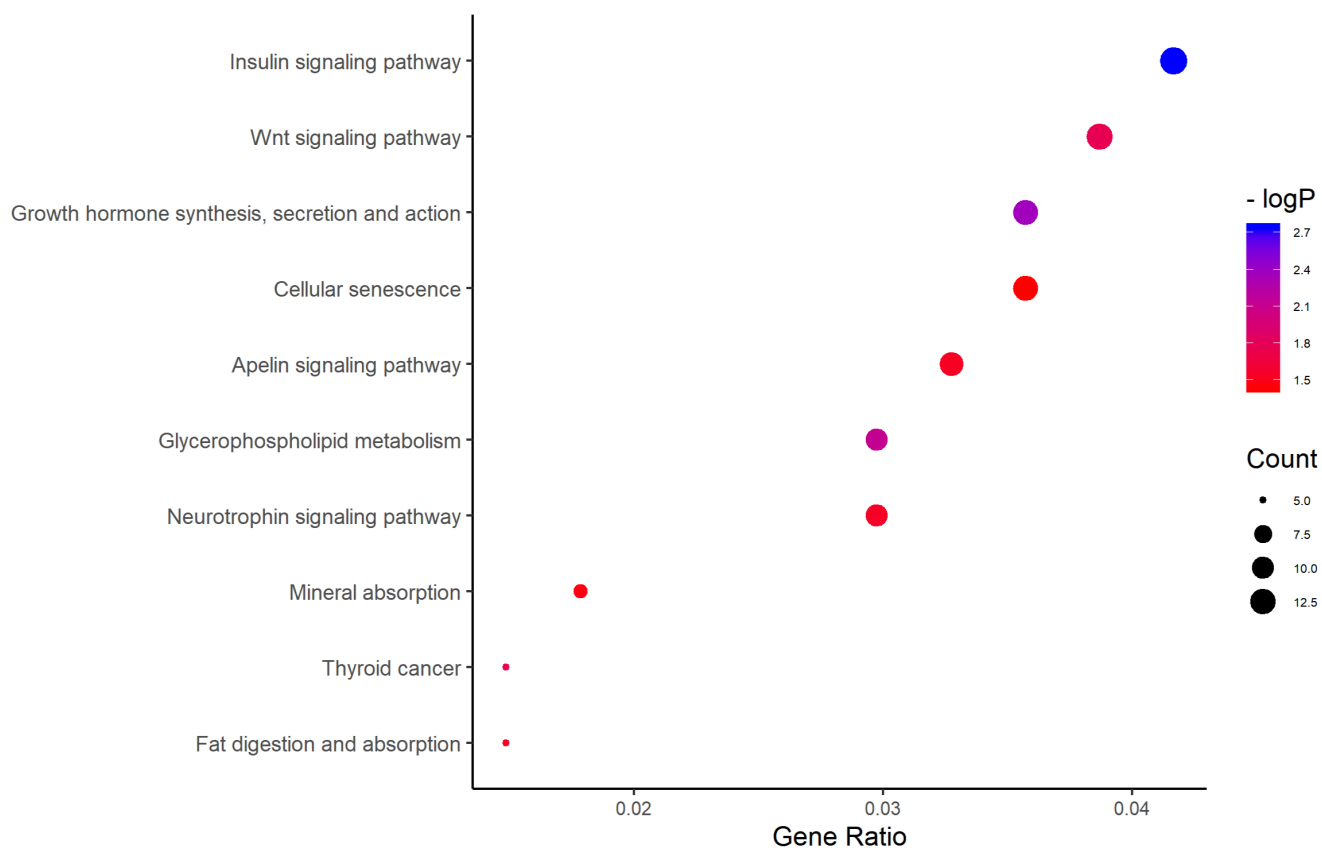

Supplement: Supplementary Materials — Supplementary Figure 1. KEGG analysis of the differentially expressed lncRNAs. Supplementary Table 1. List of the primer sequences for PCR. [file 8058770.f1.zip › 8058770.f1/Supplementary Figure S1.pdf]
